# Spatial Analysis of “Crazy Quilts”, a Class of Potentially Random Aesthetic Artefacts

**DOI:** 10.1371/journal.pone.0074055

**Published:** 2013-09-16

**Authors:** Gesche Westphal-Fitch, W. Tecumseh Fitch

**Affiliations:** 1 Department of Cognitive Biology, University of Vienna, Vienna, Austria; CSIC-Univ Miguel Hernandez, Spain

## Abstract

Human artefacts in general are highly structured and often display ordering principles such as translational, reflectional or rotational symmetry. In contrast, human artefacts that are intended to appear random and non symmetrical are very rare. Furthermore, many studies show that humans find it extremely difficult to recognize or reproduce truly random patterns or sequences. Here, we attempt to model two-dimensional decorative spatial patterns produced by humans that show no obvious order. “Crazy quilts” represent a historically important style of quilt making that became popular in the 1870s, and lasted about 50 years. Crazy quilts are unusual because unlike most human artefacts, they are specifically intended to appear haphazard and unstructured. We evaluate the degree to which this intention was achieved by using statistical techniques of spatial point pattern analysis to compare crazy quilts with regular quilts from the same region and era and to evaluate the fit of various random distributions to these two quilt classes. We found that the two quilt categories exhibit fundamentally different spatial characteristics: The patch areas of crazy quilts derive from a continuous random distribution, while area distributions of regular quilts consist of Gaussian mixtures. These Gaussian mixtures derive from regular pattern motifs that are repeated and we suggest that such a mixture is a distinctive signature of human-made visual patterns. In contrast, the distribution found in crazy quilts is shared with many other naturally occurring spatial patterns. Centroids of patches in the two quilt classes are spaced differently and in general, crazy quilts but not regular quilts are well-fitted by a random Strauss process. These results indicate that, within the constraints of the quilt format, Victorian quilters indeed achieved their goal of generating random structures.

## Introduction

Human ornaments and decorative art represent a class of biologically generated patterns typified by a high degree of structure and order. Conventional decorative patterns can typically be described by their underlying symmetry [1]. Human visual artefacts very rarely intentionally violate ordering principles such as symmetry and repetition. Although randomness serves as the typical null hypothesis in the physical sciences, it has long been known that humans have great difficulty in producing random output. Seemingly random behaviours are not uncommon in the biological world (e.g. prey escape behaviours), yet analyses of such behaviours remain for the most part qualitative [2]. It has been shown experimentally that, given the task of creating random numerical arrays, humans generate output that deviates strongly from a truly random array, especially when a participant’s response time is limited [3], [4]. Somewhat surprisingly, when participants are presented with both random numerical sequences and pseudo-random sequences produced by humans that deviate from true randomness, the latter are more likely to be classed as “random” than the truly random sequences [5]. Apparently, humans are well equipped to detect and create ordered structures, but not random structures. At least in humans, this inability seems to stem from a strong “sense of order”, a term coined by E. H. Gombrich to express how our drive to “regularise” artefacts is a fundamental aspect of human cognition, almost as basic as our sense of smell or touch [6].

Given our species’ apparent obsession with order, we might wonder if *any* human artefacts produced with a maker’s controlled actions (rather than by an uncontrollable physical process such as cracks or decay, or by minimal, uncontrolled variation) can be adequately described with a random process.

**Figure 1:**
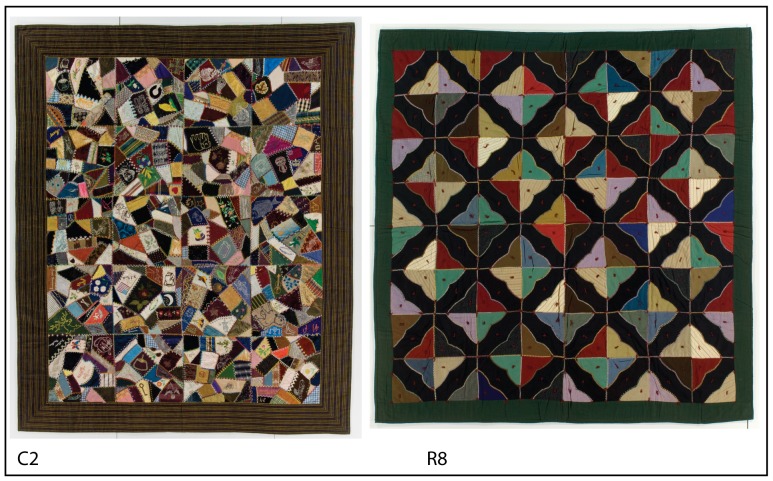
Examples of the quilts analysed in this study. Left: a crazy quilt (C2, International Quilt Study Center, University of Nebraska-Lincoln, 1997.007.0552). Right: a regular quilt (R8, International Quilt Study Center, University of Nebraska-Lincoln, 2003.003.0212). In all images, the margins that did not contain patchwork were cropped out prior to analysis.

One candidate class are crazy quilts: a once popular class of textile craftwork often intended for display. A crazy quilt is a blanket consisting of two fabric layers. The top layer is made of “irregular bits and pieces [of fabric] strewn in a seemingly disorganized fashion” [7]. This quilt type is unusual because it is made, unlike most other quilts, specifically to create an irregular aesthetic impression. It is often claimed that the arrangement of the patches is random, e.g.: “The patchwork was constructed by stitching random patches to a fabric base” [8]. In this paper, we aim to evaluate this claim by describing the properties of spatial patterns in quilts and to quantify the differences in orderedness between regular and crazy quilts (see Fig. 1 for typical examples of both quilt categories).

Our analysis of real-life visual patterns follows an approach in empirical aesthetics first outlined by Gustav Fechner in the late 19th century [9], [10]. Fechner advocated the use of three methods to investigate aesthetic proclivities in humans: studying how people produce artefacts, how artefacts are perceived and the description of properties of artefacts encountered in real life. Research on the human production and perception processes of visual patterns in the lab [11], [12] has shown that abstract geometrical patterns have a near universal aesthetic appeal and that the ordering principles underlying them are readily understood by a wide range of humans. Formal descriptions of real-life patterns, in particular Islamic tilings, exist that are based on classification systems derived from crystallography [1], [13]–[15], but very little work has been done to formally describe disorderly artefacts and patterns that do not adhere strictly to conventional symmetry classes.

Previous research applying spatial analysis tools to human artwork has focussed mainly on paintings [16]–[21], photography [22], [23] or on traditional patterns used in pottery and other ornamental objects [1], [24]. Much less quantitative research has been devoted to patchwork, though see [25], [26].

### A Brief history of Crazy Quilts

Patchwork is the stitching together of small pieces of cloth (*patches*), into a larger unit, typically used for blankets, pillowcases or clothing. Though patchwork is best known from English-speaking cultures, in particular North America and England, it is has traditionally been produced in many countries (e.g. China, Pakistan, India, Thailand, Iran, Sudan, and Korea) [8].

Crazy quilts are a form of patchwork that enjoyed a brief period of popularity in the late 19th and early 20th century. The oldest examples are from the 1870s. These quilts were widely produced until the 1920s, after which their popularity waned, although they are still occasionally produced today by patchworkers around the world.

Crazy quilts typically contain many different fabric types and fabric patterns. Additionally, the edges of patches are decorated with a wide variety of embroidery stitches and centres are often embroidered with vignettes of animals and plants, although the embroidery seems to have become less elaborate as the fad progressed [25]. In combination, crazy quilts evoke an impression of lavishness and wild abundance that stands in stark contrast to the strict rules of traditional quilts and Victorian society more generally.

The roots of Western crazy quilts may lie in Japanese patchwork. In Japan, the technique of *yosegire* (reusing precious fabrics in coats and kimonos) was popular in the 19th century. Examples of *yosegire* patchwork appear quite unstructured, lacking the rigid repetitions of Western patterns. Japan began trading with the West in 1854 with the convention of Kanagawa, ending 200 years of isolation policy. In 1876, a range of textiles were displayed at the Japanese stand of the Centennial Exposition in Philadelphia, which had close to ten million visitors (the population of the United States at the time was about 38 million) [27]. Several historians claim this exhibition and ensuing popularity of Japanese craft was the inspiration behind American crazy patchwork [8], [28]. Brick [7] also argues for a Japanese influence, but credits the Gilbert and Sullivan opera “The Mikado” which debuted in 1885, after which Japanese designs and textiles, including *yosegire* style patchwork, became wildly popular.

The oldest attested usage of the adjective “crazy”, meaning “full of cracks”, dates from the 1580s, and it is still in use today, e.g. “crazed glazing”. The contemporary meaning of “mad, insane” is attested since 1617, however evidence for describing objects or actions as mad only goes back to 1855. The oldest usage of “crazy patchwork” is found in 1885, and the appellation “crazy quilt” goes back to 1886. We therefore assume that the term “crazy quilt” at the time would primarily have meant “haphazardly cracked”, though the connotation of madness may have been present as well [29].

Crazy quilts seem to have been a rare outlet for women to escape the confines of household routines and explore individual creative expression, in some cases to the annoyance of their husbands, as the anonymous poem “The Crazy-Quilt” from 1890 suggests [30]:

[…] And where is the wife who so vauntingly swore.

That nothing on earth her affections could smother?

She crept from your side at the chiming of four

And is down in the parlor at work on another.

Your breakfasts are spoiled,

And your dinners half-boiled,

And your efforts to get a square supper are foiled

By the crazy-quilt mania that fiendishly raves,

And to which all the women are absolute slaves […].

### Spatial Analysis of Patterns

In the current study we analyse crazy quilts using spatial statistics, comparing them to `normal’ regular quilts. Quilts in general are subject to a number of constraints that would be difficult to capture in standard random models (e.g. in a Poisson process). Two key constraints are that patches must exceed some minimum size (*minimal area constraint*) and that, although rare exceptions exist, the overall quilt shape must be approximately rectangular (*edge constraint*).

In general, patch edges are straight (for the practical reason that straight seams are easier to sew than curves if the patches are to lie flat). Unfortunately, the direct analysis of patch edges as line segments (number, angle, etc) is difficult, because the seams are not necessarily perfectly straight and thus vertices and corners cannot always be unambiguously classified. In this study, we focussed instead on the properties of the patch areas and centroids, which can both be precisely calculated and serve as adequate measures of spatial organisation for our purposes.

With these constraints in mind, we adopted a Strauss process over patch centroids as our random comparison model. A Strauss process, introduced by David Strauss in 1975 [31], is a superset of a Poisson process which models interactions between points in the plane (i.e. it is a pairwise interaction process). Strauss processes were further developed by Kelly and Ripley [32] and have been applied to a wide variety of biological spatial patterns, for example to model herd animal dispersion [33], spatial distribution of tree species [34] or neuron locations in the brain [35].

Because of imprecision in manual motor control, imperfection is inherent in any handmade object. To estimate the magnitude of this intrinsic motor error, and to provide a rigorous basis for comparison, we also analysed standard or `regular’ quilts. In this type of quilt, multiple copies of the same pattern unit (`blocks’) are arranged in a translational fashion on a square grid. While each block is a nearly exact copy of the pattern, blocks will nonetheless show some unintentional random variation. Minimally, we predict that crazy quilts will be significantly more random than such regular quilts. A finding that qualitatively different spatial models fit these two classes would be germane to our overall question of the hypothesised randomness of crazy quilts.

### Hypotheses

Our overall goal in this study is to evaluate the degree to which crazy quilts are compatible with a random generative process, and the degree to which this differentiates them from regular quilts. This broad question leads directly to testable hypotheses concerning patch area and patch centroid location (labelled HR and HC for hypotheses about regular and crazy quilts, respectively):

HC1: Crazy quilts are intended to create a haphazard and irregular impression. If this intention is realized, the location of patch centroids should be adequately modelled by a random spatial process.

HC2: Because crazy quilts lack repeating motifs or patch types, the patch areas should come from a single overall distribution. Furthermore, as patch ensembles are constrained to fit within rectangles, we expect small patches to be more numerous than large ones. We thus predict a positive-skewed but otherwise continuous distribution of patch sizes.

HR1: Because patterns in regular quilts are intended to be periodic and symmetrical, the locations of patch centroids should not be adequately modelled by a random spatial process.

HR2: Regular quilts are made up of repeating motifs consisting of a small number of patch types. Because each element of a given type is intended to be identical in size and shape, but will include some small degree of error, we expect the overall patch size distribution of a regular quilt to be a composite of the individual distributions for each patch type as a mixture of Gaussian distributions (rather than the single overall distribution predicted for crazy quilts, in HC2).

## Materials and Methods

We performed a detailed spatial analysis of hand-tracings of 8 crazy quilts and 8 regular quilts from North America. Their overall properties are summarised in table 1. To ensure that the quilts had a comparable level of structural complexity and similar internal constraints, all quilts had at least one level of regular subdivision, i.e. were organised either in regular blocks or strips. Because many quilts were made anonymously, it was not possible to date the quilts exactly, but based on the published sources, we ensured that the quilts stemmed from roughly the same geographic area (USA) and time (ca. 1870–1930). Additionally, we only selected images that showed the entire quilt in sufficient detail to allow an exact delineation of patches within the quilts. The analysed quilts were selected from commercially available quilt books [7], [36], [37]. In general, these quilts were blanket size, but one of the crazy quilts (C5), was considerably smaller than the others, roughly pillowcase size.

**Table 1:** Overview of the quilts analysed.

Quilt	Year	Number ofPatches	Height(in cm)	Width(in cm)	Overall area(in cm^2^)	Patched area(in cm^2^)
R1	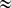 1930	784	208.28	208.28	43,381	15,661
R2	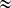 1930	440	203.2	175.26	35,612	17,934
R3	1898	421	218.44	165,1	36,064	18,520
R4	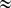 1930	327	215.9	209.55	45,241	31,877
R5	1891	972	187.96	173.99	32,703	32,020
R6	1890–1910	692	191.77	187.96	36,045	19,980
R7	1890–1910	736	201.93	200.66	40,519	25,460
R8	1900–1920	192	182.88	173.99	31,819	25,948
C1	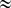 1930	239	198.12	160.02	31,703	21,895
C2	1871	512	193.04	160.02	30,890	21,327
C3	1884	317	207.01	180.34	37,332	35,757
C4	1885	834	195.58	162.56	31,793	31,793
C5	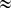 1875	108	35.56	35.56	1,265	1,203
C6	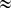 1890	322	139.7	200.02	27,943	26,636
C7	1880–1900	106	134.6	132.08	17,778	17,505
C8	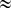 1889	133	210.82	170.18	35,877	34,550

Size and numbers of patches of the quilts analysed and the exact or approximate year of production. We excluded border stripes from the size measurements in our analysis, including only the region that contained patchwork (“patched area”).

**Figure 2:**
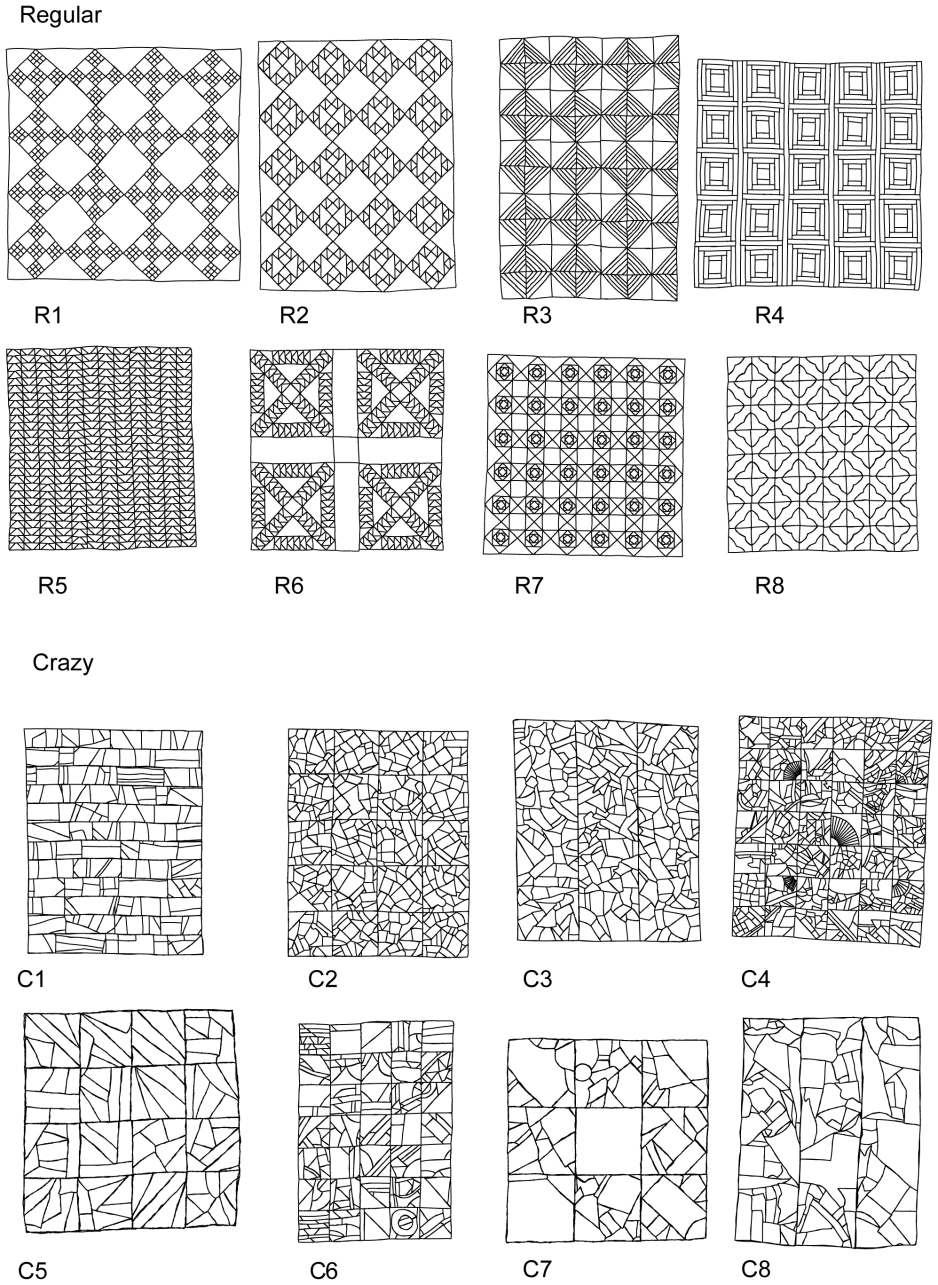
Patch outlines of the sixteen quilts that were analysed (borders were not analysed and are not shown). R1–R8: “Regular” quilts with traditional repeating geometric patterns. C1–C8: “Crazy” quilts with no obvious repeating pattern.

Digital images of the quilts were scanned from printed photographs at 300 dots per inch (CanoScan LiDe 200, Canon). Despite intensive efforts, we were unable to use segmentation algorithms to derive accurate patch borders automatically, due to considerable internal complexity and heterogeneity of the quilt patches. Thus, the outlines of individual patches were traced manually on a Wacom LCD tablet (DTZ-1200W/G) and saved as regions of interest (ROIs) using FIJI [38]. The tracings are shown in Fig. 2. We converted the ROIs from pixels to cm^2^ by scaling the scanned image based on the measurements of the photographs of the quilts and the dimension given in the source books. This scale was estimated twice, based on length and width measurements, and then averaged. Unless indicated otherwise, “area” refers to true area, in cm^2^, hereafter. We excluded non-patched borders (long continuous strips of fabric) from the analysis, isolating the area containing patches by using the smallest possible bounding box around the patched area. For each individual patch, we computed the centroid (by averaging the x and y values of the pixels within the patch) and the area in FIJI. Measurement accuracy was evaluated by remeasuring one randomly selected patch from each quilt ten times and analysing the absolute range of measurements for each quilt category.

We analysed the patch area distributions in two ways: first, by fitting multimodal distributions (Gaussian mixture models) and second, by fitting various standard random unimodal distributions. For unimodal distributions, we chose three plausible candidates that take only positive values: the gamma, Weibull and lognormal distribution, with no *a priori* reason to favour any one of these particular distributions.

The gamma distribution can take a wide variety of forms, which has led it to be widely used for modelling spatial and temporal characteristics of rainfall [39], mutation rates in human mitochondrial DNA [40] and rate of material deterioration [41].

The Weibull distribution [42] is often used to model product failure, but also human aging and mortality (for a review of recent applications see [43]). Furthermore, the distribution of two- and three-dimensional particle sizes, e.g. airborne dust particles, can also be well-modelled with the Weibull distribution [44], [45].

The lognormal distribution applies to variables whose logarithms have a normal distribution [46]. While widely used in biological modelling [47], [48], it has been suggested by Brown and Wohletz [49] that although the Weibull and lognormal cover data similarly, the Weibull is more empirically grounded in the case of fragmentation of particles into smaller particles and that “the empirical use of the lognormal distribution for particle size studies over the last century may have been simply fortuitous” ([49], p.15).

Finally, we also included the standard normal distribution, since we predicted this would fit regular quilts best (in the form of Gaussian mixture models).

We used maximum likelihood estimation to fit the distributions with the R (version 2.12.2, http://cran.r-project.org/) package “fitdistrplus” (version 0.3–4). We scaled the area values down (area×0.01) as required to bring values into the supported distribution range [50] for all quilts except C5, where we used area×0.1 because the quilt was smaller than the others and required less reduction. We used the Akaike Information Criterion (AIC) [51], a measure derived from the log likelihood function, to assess which of these distributions fit the data best. We considered all top-ranking candidates (with a difference in AIC values (ΔAIC) less than 2) to be likely candidate distributions [52]. To additionally evaluate the likelihood of one model over the other, we follow Burnham and Anderson [52] in converting AIC values into normalised Akaike weights which indicate the likelihood of a model given the data. This adjustment is particularly useful when comparing two models with similar AIC values. Unlike conventional statistical tests, AIC does not allow *absolute* inferences about how well a model fits the data; instead it provides a *relative* assessment of which of the available models fits the data best, compared to the other candidates.

We constructed Gaussian mixture models for both quilt categories. For regular quilts, we classified the patches into categories (i.e. the different squares, triangles etc that occurred in the pattern block) manually. We used the area means, standard deviations and relative frequencies of each patch category to seed the mixture models which we then used to randomly generate the same number of elements as in the quilts. Initially, we constructed mixture models with patch categories estimated by the Bayesian Information Critierion and no external seeding of category information, which led to higher rates of misclassified patches, since pattern elements may have different shapes, but similar areas. We thus opted for seeded models that offered comparably good fits and reflected the number of motifs in regular quilts accurately.

Using seeded models, we plotted the actual patch area distribution and the distribution of the estimates of simulated patch category areas using the R package “mixtools” [53]. For crazy quilts, we used the identical procedure, but we used the overall mean and standard deviation of the whole dataset of each quilt as seeds, since there were no obvious patch categories. We used bandwidth values (which are equal to the standard deviation of the kernel estimates) as a proxy to test for the difference in the amount of smoothing required to fit the distributions in the two quilt categories.

To test the goodness of fit of these Gaussian models explicitly, we generated 39 simulations with the model parameters derived from the data using the R package “mixtools” and custom Python software (version 2.65, http://www.python.org). The area values of the simulations and the actual data were sorted by size, and the minimum, maximum and actual areas were then plotted. If the actual areas were above the maxima or below the minima generated by the simulations, we interpreted this as a significant deviation (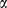 = 2/40 = .05) from the model.

We analysed the skewness of the area distributions with the R package “moments” [54]. Again, for crazy quilts, we used the overall distribution, but for the regular quilts, we analysed skewness for each of the patch categories separately.

Moving from patch area analysis to the spatial distribution of patch centroids, we fitted Strauss models to the patch centroids of both crazy and regular quilts in R using the package “spatstat” [55]. Strauss processes model the random spatial distribution of points that do not overlap or coincide. The parameter *r* of the Strauss process denotes an interaction distance between points. This parameter must be larger than zero, to satisfy the “no overlap “ constraint. The parameter 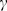 controls the strength of the interaction between points. If 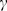 = 1, then the process is a Poisson process with intensity 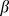 (average number of points within a certain area), whereas if 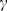 = 0, then the process is “hard core”, that is, the points can never lie closer together than distance *r* [56]. Thus, 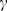 describes the interaction between the points, and *r* describes the distance in which this interaction can occur. The goodness of fit of the Strauss process can be assessed by the L-function, which is based on Ripley’s K-function [57]. The K-function counts the number of occurrence of points within varying distances (*r*) around each point [58]. For complete spatial randomness, *K(r)* = 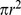. The *L* value is a transformation of the *K* value: *L* = (*K*/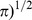.

*L* is preferable to *K* for our analysis because it is constant in a Poisson pattern (*L* = *r*), unlike *K*. That is, transforming *K* to *L* removes the contribution of the random Poisson process from the distribution, showing only the effects of *r*. Because we had no *a priori* reason to believe that a Strauss process was specific to either quilt category, we applied this process to both regular and crazy quilts. To estimate the value of *r*, we applied the method of maximising pseudolikelihood [56]. This approach was originally proposed by Besag to estimate the unknown parameters of a sample that do not follow a multivariate normal distribution [59], [60]. We tested all values between the minimum and maximum interpoint distances (*r*) in 0.01 steps. The value with the maximum pseudolikelihood was chosen as the optimal interaction radius *r* for the model of the Strauss process fitted to our patterns. The highest and lowest of the simulated values form simulation envelopes that determine the critical points (i.e. 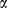 = .05) of the Monte Carlo test for upper and lower *K* values [57], [61].

To estimate the effect of the border on the patterns (for example, the centroids might be more sparse near the quilt edges), we ran the process twice, with and without an isotropic border correction, and compared the resulting *r* values. For each quilt, the goodness of fit of the parameter was then tested by 39 simulations of *N* random points (with *N* = number of centroids present in the original quilt), placed randomly in a space of the same dimensions as the quilt, constrained only by the parameter *r*. For those cases where there was no effect of the isotropic correction, we also ran the simulations of the model fit without any corrections, using the estimated *r* value. With two exceptions (C2 and C4), the values for *r* estimated with and without isotropic border correction were identical. This implies that the effect of the border on centroid distribution is weak. For the two exceptions, we ran the simulations both with and without the isotopic correction and compared the fit. For C2, the fit of the simulation was the same with either *r* value, while it improved for C4 with the correction.

In addition to the R packages already mentioned, basic statistical analyses were performed in SPSS version 17 (http://www-01.ibm.com/software/analytics/spss) and using custom Python scripts.

## Results

### Basic Quilt Statistics

In total, the quilts contained 7,135 patches (regular: 4,564, crazy: 2,571). Crazy quilts had on average 321 patches (range: 106–834), while regular quilts had on average 571 patches (range: 192–972) but this apparent trend for more patches in regular quilts did not attain statistical significance (Mann-Whitney U test: p = 0.06, U = 14,000). There was no obvious relationship between number of patches and quilt size for either quilt type (Linear, logarithmic, inverse, quadratic and cubic regressions were attempted, all p>.396). The total patched area in cm^2^ for crazy quilts averaged 23,102 (SD: 10,871) versus 23,425 (SD: 6,349) for regular quilts, which was not statistically significant (Mann-Whitney U test: p = 0.64, U = 27.5). Thus, the quilt categories were comparable with regard to size, number of patches, and no relationship was found between number of patches and overall quilt size for either category. The difference in manual measuring error for the two quilt types (Crazy quilts: 3.25% measuring error, SD: 2.38. Regular quilts: 3.35% measuring error, SD: 1.86) was not statistically significant (Mann-Whitney U test, p = .8, U = 29).

### The Distribution of Patch Areas

Hypotheses HC2 and HR2 predict significant differences between the two quilt types in their distributions of patch sizes. We found that the patch areas for regular quilts were indeed characterized by multimodal distributions, while the distributions for patch areas of crazy quilts were unimodal. As predicted, the overall area distributions of the crazy quilts had a strong positive skew (mean skewness: 2.3, SD: 1.4). In the case of regular quilts, the patch area distributions of each patch category were only weakly skewed and were split between positive and negative skew: 20 categories had a negative skew (mean skewness: −0.74, SD: 0.83) and 24 had a positive skew (mean skewness: 1.46, SD: 2.11), suggesting that variation in patch areas due to variation in motor control is not *a priori* skewed either way. In summary, we found that the patch area distributions of crazy quilts were unimodal, while the patch area distributions for regular quilts were multimodal, confirming HC2 and HR2.

### Crazy Quilts: Unimodal Distribution Types

**Figure 3:**
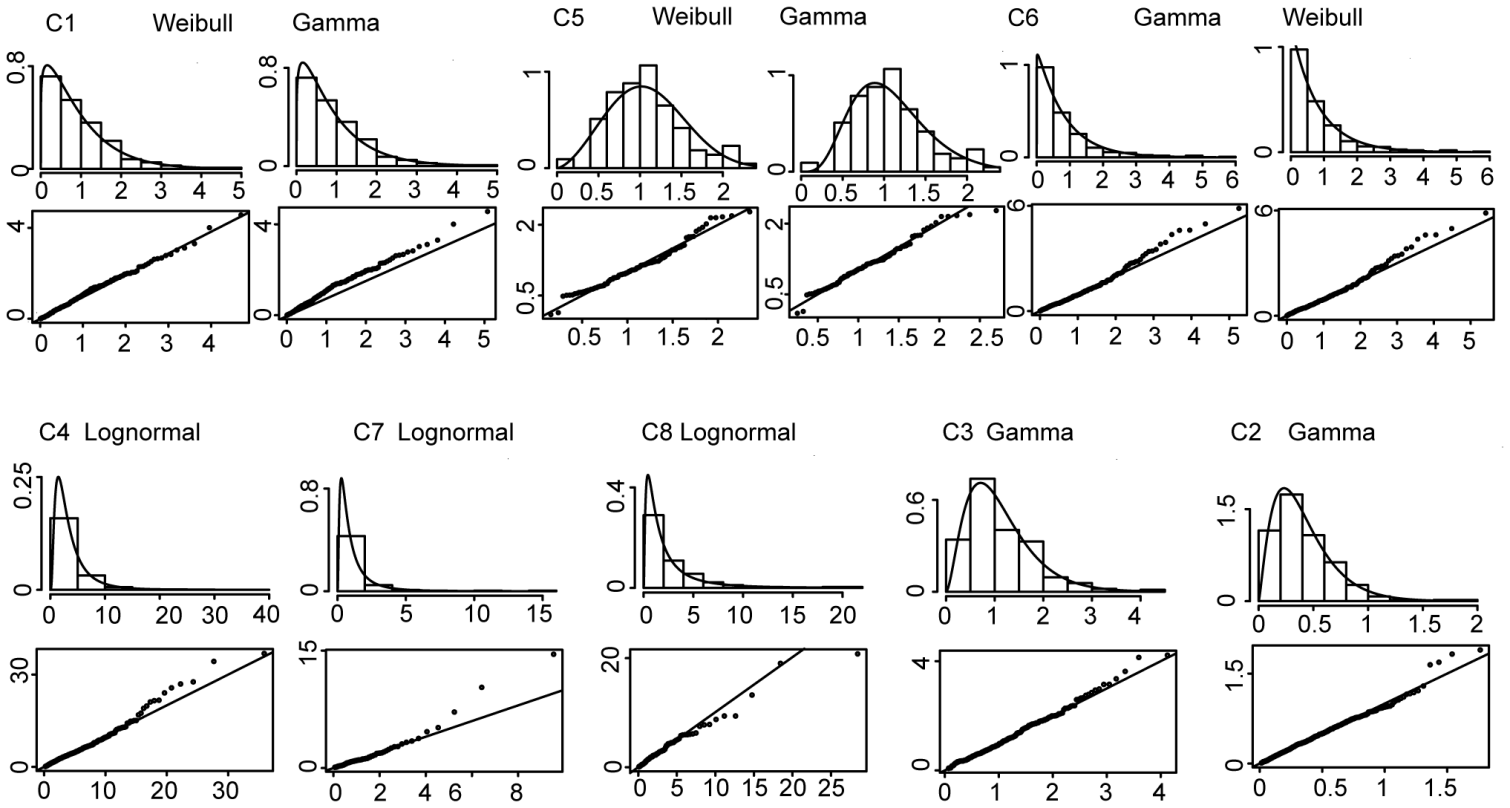
Random distributions fitted to the crazy quilt patch areas. In those cases where ΔAIC<2, both distributions are shown. For each quilt, the best fit distributions and the histograms of the area distributions are superimposed in the top graph. Below, the QQ plots of the quilt sample quantiles (X-axis) and the theoretical quantiles as predicted by the fitted distributions (Y-axis) are shown.

The unimodal distributions underlying different crazy quilt patches did not consistently fit with a single distribution type. As expected, due to the constraints of quilt-making, the normal distribution was a very unlikely candidate for all cases. In three cases, lognormal clearly was the best candidate, with no other distributions being very likely (all ΔAIC >9.42). The AIC values for gamma and Weibull distributions in general were much closer: in three cases, ΔAIC was <2, so either of these two distributions provide a possible best candidate (see table 2). The gamma distribution was the strongest candidate in two further cases (C2 and C3), where no other candidate distribution was likely. However, in all those cases where the Weibull distribution was a likely candidate, the gamma distribution was also likely, and the Akaike weights for the Weibull distribution were not very strong, not exceeding a probability of 70%. Overall, we observed a split between the crazy quilts where lognormal was the best candidate (N = 3) and those cases in which gamma and Weibull fit best (N = 5). Figure 3 shows an overlay of histograms and the best fitting distributions as well as QQ plots of the theoretical distributions and the actual data. Deviations of the data from the theoretical distributions are most visible in the high quantiles, which is unsurprising, because there are fewer large patches than small patches in the quilts, and thus the data is sparser in the high quantiles. In summary, crazy quilt patch area distributions were well-modelled by various distribution classes but no single type fit all exemplars.

**Table 2:** Unimodal distributions fitted to crazy quilts.

Quilt	C1	C2	C3	C4	C5	C6	C7	C8
Gamma	**1.72 (30)**	**0 (.99)**	**0 (.90)**	81.89 (<.01)	**1.86 (.25)**	**0 (.51)**	29.44 (<.01)	9.56 (<.01)
Weibull	**0 (.70)**	8.53 (<.01)	15.97 (<.01)	140.86 (<.01)	**0 (.64)**	**0.18 (.49)**	28.89 (<.01)	9.42 (<.01)
Lognormal	53.41 (<.01)	48.49 (<.01)	4.39 (.10)	**0 (1)**	17.95 (<.01)	25.58 (<.01)	**0 (1)**	**0 (.98)**
Normal	125.34 (<.01)	165.61 (<.01)	107.18 (<.01)	872.04 (<.01)	3.45 (.11)	324.41 (<.01)	204.52 (<.01)	172.08 (<.01)

AIC values for the different distributions fitted to the quilts. The best fitting distributions are marked in bold, with the Akaike weights given in brackets. If ΔAIC<2 for two models, we considered both models to be a possible fit (this was the case for quilts C1, C5 and C6).

### Regular Quilts: Gaussian Mixture Models

**Figure 4:**
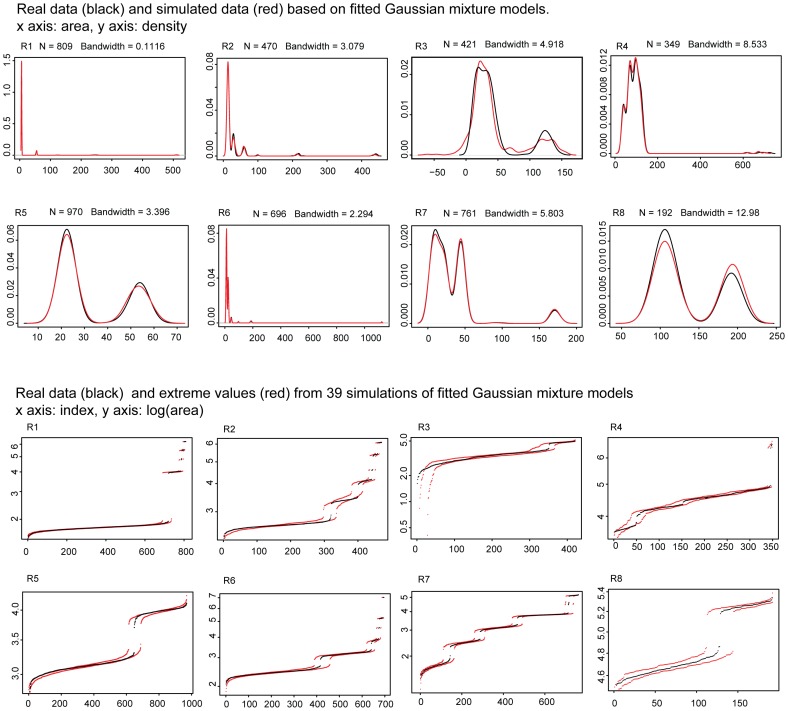
Top panel: The kernel density estimates of Gaussian mixtures of patch areas of regular quilts. The black line shows the actual data and the red line shows the the Gaussian mixtures simulated based on patch categories. Bottom panel: Fit of the data (black) and 39 simulations (highest and lowest values of the simulations indicated with red dots (log scale).

**Figure 5:**
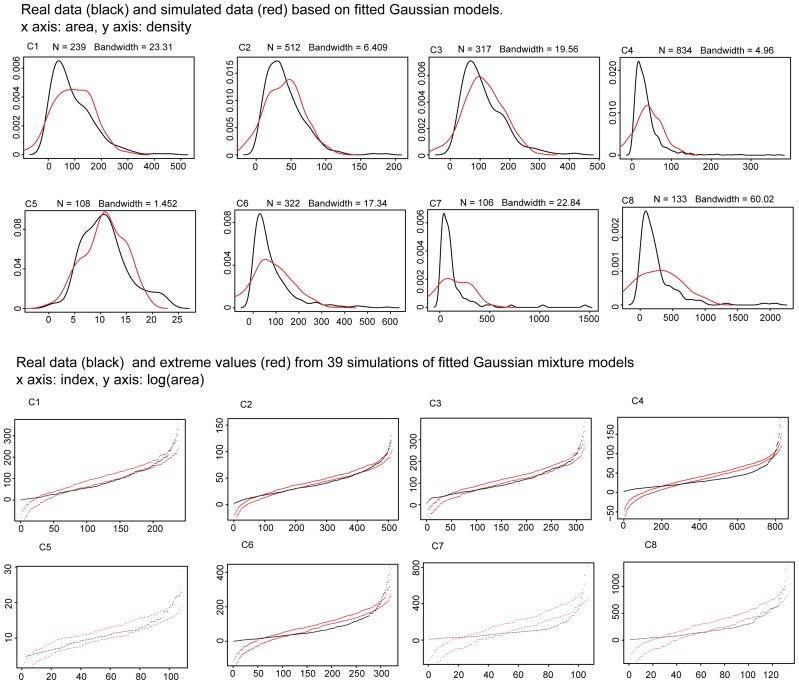
Kernel density estimates of single Gaussian distributions of the patch areas of crazy quilts. The black line shows the actual data and the red line shows the Gaussian mixtures simulated based on patch categories. Bottom panel: Fit of the data (black) and 39 simulations (highest and lowest values of the simulations indicated with red dots).

In contrast, kernel density estimates of Gaussian mixture models proved very good fits to the multimodal patch area distributions in the regular quilts (see Fig. 4), while the estimates of Gaussian models for the crazy quilts (see Fig. 5) show little overlap with the estimates of the real distributions. In particular, the Gaussian models for crazy quilts extend into negative values, violating our minimal size constraint. Standard Gaussian distributions thus provide poor models for crazy quilts.

The difference in bandwidths (standard deviation of the kernel density) for the two quilt types was statistically significant (Mann-Whitney U test: p = .03, U = 12, Cohen’s *d* = 1.075) and much higher for crazy quilts (mean bandwidth for regular quilts: 5.14, for crazy quilts: 19.49), indicating that a significantly higher degree of smoothing was required for a Gaussian distribution to be even approximately fitted to crazy quilts.

We calculated the number of occurrences when the actual patch area values were above the maximal value or below the minimal value of the simulations (see Table 3). The differences in the percentage of deviations between the two quilt types was statistically significant (Mann-Whitney U test: p<.001, U = 64, Cohen’s *d* = 3.36). Thus overall, Gaussian mixture models proved a significantly better fit for the regular quilts (Fig. 4) than the crazy quilts (Fig. 5), based both on bandwidth differences and the fit of the simulation envelopes.

**Table 3:** Deviations from Gaussian mixture models for crazy and regular quilts.

RegularQuilts	# of deviations(%)	CrazyQuilts	# of deviations(%)
R1	0 (0)	C1	119 (49.79)
R2	156 (33.19)	C2	329 (64.26)
R3	21 (4.99)	C3	179 (56.47)
R4	7 (2.00)	C4	719 (86.21)
R5	173 (17.84)	C5	5 (4.63)
R6	9 (1.29)	C6	249 (77.33)
R7	0 (0)	C7	77 (72.64)
R8	0 (0)	C8	84 (63.16)

Deviations above or below 95% limits of Monte Carlo simulations based on Gaussian mixture models for regular and crazy quilts. Both the number of deviant patches, and their corresponding percentage of the total number of patches in that quilt, are given.

### Spatial Distribution of Patches: Fitted Strauss Processes

The previous results show that there are fundamental differences between crazy and regular quilts in terms of the distributions that best describe patch areas. We also evaluated the degree to which a well-defined random process – a Strauss process – can be used to model patch centroid locations for the two quilt types.

**Figure 6:**
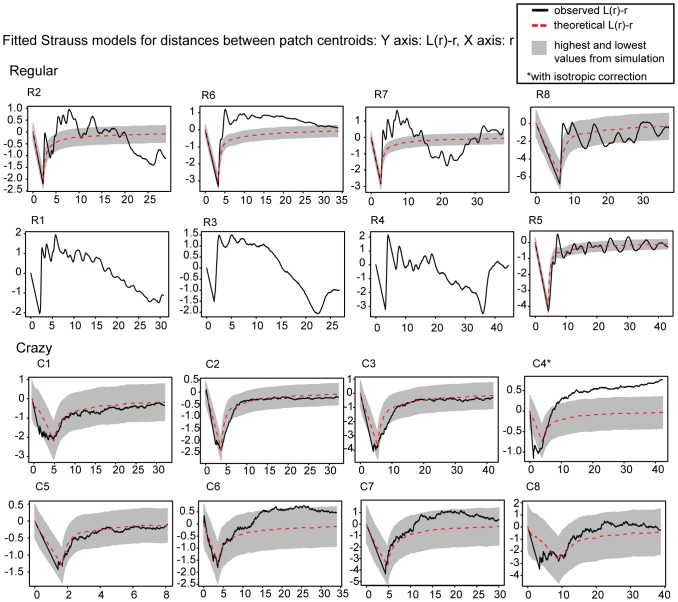
Simulation envelopes of fitted Strauss models for regular quilts (top panel) and crazy quilts (bottom panel) based on centroid locations. For three regular quilts the simulation algorithm did not converge and thus no simulation envelope is shown. The solid black line depicts the actual data, and the data predicted by the model is shown with a dashed red line. The grey envelopes show the highest and lowest values of 39 simulations. The Y-axis shows the L-function, derived from Ripley’s K-function, and the X-axis shows the varying *r* values. A deviation of the black line beyond the grey envelope can be interpreted as a significant deviation of the data from the fitted Strauss model.

We first estimated the point interaction parameter *r* from the data from both quilt classes using maximum pseudolikelihood. We then simulated a Strauss process with points randomly placed in space, under the constraint of this *r* estimate, and compared them with the actual distributions. The results of the simulations are shown in figure 6: density values are plotted on the X axis, and the *L* values for various distances (*r*) are plotted on the Y axis (roughly, *L* gives number of other points lying within the distances of a focal point, see Methods). The simulation envelopes based on the highest and lowest ranking values from 39 simulations are shown as grey bands, while the dashed red line shows the predicted *L* values of a fully random Strauss process. For quilts accurately modelled by such a process, the data (black line) should be within the grey envelope. A deviation of the `observed’ line above the envelope means that there are more points within the Strauss interaction radius *r* than predicted by the model, and a deviation below the envelope that there are fewer points than predicted, i.e. that there is repellence between the points.

When fitting the Strauss process with the estimated *r* values, the model fitting function returned 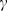 values 1 for three of the eight regular quilts (R1, R3 and R4). As the Strauss process is only defined for 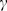 values ≤ 1, this is strong evidence that it is not an appropriate model for these centroid sets. Therefore, we did not fit Strauss processes to these quilts, but do show the density of centroids for various *r* values for these quilts in figure 6. In the graphs, a fully random pattern with no interpoint interaction (i.e. Poisson) would be a straight line. Clearly, the distributions of patch centroids deviate strongly from a Poisson process. However, for crazy quilts, in contrast to regular quilts, the Strauss process typically provides an excellent fit. While C6 and C7 show some clustering at medium radius values, this is within the simulation envelopes. Only C4 was consistently and significantly under-dispersed across the whole range of *r* values. One possible reason for this is that C4 had a very large number of patches for its size, so that centroids were consistently closer together than predicted by a random model. Furthermore, C4 is unusual in that it contains two isolated regular `fan’ shapes.

Figure 6 clearly illustrates that the regular quilts cannot be accurately modelled with a Strauss process. Unlike crazy quilts, regular quilts show large oscillations in the *L* value as *r* increases. reflecting the regular clustering of patches. In sum, the distributions of centroids in crazy quilts, but not regular quilts, are generally consistent with a simple Strauss random process. These data are clearly consistent with our hypotheses HC1 and HR1.

## Discussion

Using statistical spatial analysis tools, we found clear differences between regular and crazy quilts. We showed that the distributions of patch areas differ for the two quilt categories: the patch areas of regular quilts follow a multimodal distribution, the peaks of which correspond to the patch categories of the pattern, consistent with hypothesis HR2. In contrast, patches of crazy quilts have unimodal distributions (consistent with HC2), but no single random function consistently fits the distributions best. In all crazy quilts, the area distributions had a positive skew, i.e. small patches are more frequent than large patches. These findings were consistent with hypothesis HC1 concerning the areas of patches in crazy quilts.

For the crazy quilt patch sizes, we found that the Weibull distribution and the gamma distribution were an equally likely fit in three cases and the lognormal was the best fit for three others, but there was no overlap between gamma and Weibull on the one hand and lognormal on the other. Thus different random distributions approximate the patch size distributions of regular quilts.

Concerning centroid locations, we found that patch centroids of crazy quilts could be accurately modelled by a random Strauss process with one parameter (*r*) derived from the data, consistent with our hypothesis HC1 about the essentially random placement of patch centroids in crazy quilts. Furthermore, this analysis indirectly supports our hypothesis concerning the non-random placement of centroids in regular quilts (HR1).

The results of this investigation show clearly that, despite humans’ well-documented difficulty with recognizing or generating random sequences, Victorian quiltmakers were able to intentionally produce spatial patterns compatible with random processes. The clear distinction we found between regular quilts and crazy quilts shows that the randomness observed in crazy quilts does not result from low-level motor inaccuracy, which is equally present in both quilt types. We demonstrated a close fit between quilt centroids and random Strauss processes in which the only fitted parameter was a minimal distance between patch centroids. This shows that within the constraints of the patchwork method itself (which demands a certain minimal amount of cloth simply to stitch the patches together), crazy quilt properties match those expected from a random spatial process. We thus conclude that Victorian-era quilt makers achieved a level of intentional spatial randomness that, to our knowledge, has never been documented in any other human artefact. Our results however do not allow inferences to be drawn concerning the actual production process, which obviously would not have been entirely random, and would have required some planning (e.g. adjusting size of the quilt to the available amount of fabric or planning even colour distributions). However, a description of the end product, as we have undertaken here, has the advantage that it could be applied to other artefact types. For example, we think it would be fascinating to compare these findings with other random seeming human made patterns such as crackle glazing, patchwork made in Japanese and Korean traditions, stained glass, mosaics, pavings etc, using the techniques developed here.

The results presented here do not, of course, suggest that all aspects of crazy quilts are random. Obviously, the weave of the fabric patches, or the stitches used to combine patches, are highly regular. Furthermore, individual patches were traditionally often decorated with detailed, and often representational, needlework which is anything but random. Finally, the colour selection of patches appears, at least in most cases, to be non-random (although we did not analyse colour in the current study, and accurate determination of a single “colour” for the complex fabric patches typical of our quilts is far from trivial, see [26]).

The multimodal distributions underlying the regular quilts derive from the repeated production of the same pattern motifs. The repeated production of multiple units to create symmetrical patterns may thus be the visible manifestation of humans’ unusual cognitive proclivity for order and symmetry [62]–[66].

Naturally, it would be intriguing to record and model the actual production process of crazy and regular quilts to gain insight into the levels of planning involved in producing crazy versus regular quilts. In particular, it is interesting that many crazy quilts have an intermediate level of organisation in to blocks or stripes. The organisation of a production process into discrete chunks offers advantages in terms of efficiency [67]. Examining how hierarchical organisation benefits the quiltmaking process, which is guided not only by efficiency, but also by aesthetic considerations, may offer further insights into the organisation underlying human self-guided productive processes more generally.

The details of the process by which historical crazy quilts were produced are unavailable today, although it would be possible to document the generation process in present-day quilt makers. More practically, it should be possible to mimic key features of quilt making (e.g. patch selection, trimming and combination processes) with computer interfaces to investigate aspects of the quilt-making process in the laboratory. We see no reason to doubt that any human provided with such an interface could produce random patterns like those documented in our quilts, given that, in their heyday, crazy quilts were produced by a substantial proportion of quilters. Such a research project based on Fechner’s “method of production would be a logical, and we think valuable, extension of the analyses reported here.

Quilting remains an extremely popular tradition. Crazy quilts are well known, but rarely made today. The highly ordered and hierarchically-structured regular quilts represent a much older, and much more persistent, patchwork tradition. It is unlikely that this dominance results from a practical or economic constraint, since it would be much easier to turn a bag of cloth scraps into a crazy quilt than a regular quilt, and the measurements and straight lines required to make a regular quilt are both more difficult, and more wasteful of cloth, than those needed to create a crazy quilt. Instead, we suggest that the rarity of crazy quilts and their short-lived popularity provides a clear historical indicator of the deep, and as yet unexplained, drive in our species to surround and adorn ourselves with structured and symmetrical, rather than random patterns: an evocative reflection of what Gombrich [6] termed the human “sense of order”.
